# Synthesis of an Aqueous Self-Matting Acrylic Resin with Low Gloss and High Transparency via Controlling Surface Morphology

**DOI:** 10.3390/polym11020322

**Published:** 2019-02-13

**Authors:** Qiwen Yong, Caizhen Liang

**Affiliations:** 1 Chemical Synthesis and Pollution Control Key Laboratory of Sichuan Province, China West Normal University, Nanchong 637002, China; 2 Institute of Applied Chemistry, China West Normal University, Nanchong 637002, China; 3 State Key Laboratory of Applied Microbiology Southern China, Guangdong Provincial Key Laboratory of Microbial Culture Collection and Application, Guangdong Institute of Microbiology, Guangzhou 510070, China; liangcz@gdim.cn

**Keywords:** aqueous acrylic resin, cross-linking monomer, low gloss, self-matting effect, glass transition temperature, high transparency

## Abstract

This paper reports on a novel, film-forming acrylic polymer resin that exhibits low-gloss surface and high transparency via controlling film morphology at sub-micron roughness levels. Such microstructure is controlled by means of the copolymerization process increasing the allyl methacrylate (AMA) crosslinker content from 0 to 0.4 wt %. This acrylic resin makes it possible to avoid high loadings of matting agents, while also having good abrasion resistance and soft-touch feeling. Gloss levels of as low as 4 units at 60° incident angle and light transmittance of up to 85% have been achieved. The chemical structure of the aqueous acrylic resin was characterized by ATR-FTIR and NMR spectroscopy. The film morphology and surface roughness were measured by SEM and AFM analysis. The emulsion particle morphology and glass transition temperature were obtained by TEM and DSC, respectively. The effects of the crosslinker content on the light transmittance, glass transition temperature, and thermal degradation stability were also discussed in detail. The characterization results conclude that an acrylic polymer with interesting optical properties and high thermal stability can be obtained, which is desirable for leather applications.

## 1. Introduction

Coating systems and processes which give a substrate a uniform and matt surface are of considerable interest. The reasons for this are predominantly practical in nature. In comparison with a highly glossy surface, a matt surface is superior to hide surface imperfections and does not require to be cleaned very frequently [1,2,3]. The matt surface may also be desirable on safety grounds to avoid the strong reflection of light [4,5]. The reduced glare of surfaces in schools, hospitals, and workplaces helps to minimize the visual distraction, so that people’s concentration is more focused in those environments [6].

It is universally known that aqueous acrylic polymers such as those of lower alkyl acrylates and lower alkyl methacrylates, as well as their copolymers are widely used in the coating industry [7,8,9]. However, most of the acrylic polymers dry to produce generally highly glossy surfaces [10,11]. To reduce the gloss of aqueous acrylic coatings, varieties of non-soluble particles, known as matting agents or dulling agents, are added into aqueous acrylic resins. During the drying process, the loss of solvents and condensation products result in a fairly high shrinkage of the acrylic coating film. Consequently, a diffusely reflecting micro-textured surface is formed [12,13,14]. The most common dulling agents are silica or waxes, or both of them in combination [15,16,17]. Clays, metal soaps, ground-ups, or sometimes hard organics are also used [18,19]. However, the common characteristic of these matting agents is that they are hard, inflexible materials that must be mixed with a liquid-based coating. In addition, the amount of liquid-based resin must be sufficient so that it completely envelops the non-soluble particles and fills the interspace between them. When extremely low gloss levels are demanded, use of such high levels of hard dulling agents would have the problem that they could not always be adequately wetted and surrounded by liquid-based resin, or in other words, they could not be restrained into a uniform and continuous film. In such cases, the mechanical properties of the liquid-based coating would be impaired [20]. Since the matting agents are also individual hard particles, compared to the dense structure of liquid-based resins, it is difficult to obtain a soft-touch surface [21]. Therefore, the development of an alternative method of fabricating a low gloss acrylic polymer without these drawbacks would be highly desirable.

This paper reports a novel aqueous emulsion of acrylic polymer that is inherently of low gloss. The polymer meets the main objective we established based on marketplace demand for very low gloss and high transparency surfaces when utilized as a leather finish. The key strategy of preparing the low gloss acrylic polymer resin is controlling the microstructure for yielding a tailor-made roughness. Such microstructure is achieved by controlling the amount and addition order of each monomer, as well as the introduction of a cross-linking monomer allyl methacrylate (AMA), which can cause the incompatibility within the resultant resin. This causes the formation of a coating that has a micro-rough surface rather than one that is smooth. The majority of this paper is concerned with what the chemical structure of this acrylic polymer resin is, through the FTIR-ATR and NMR spectroscopies, and what this polymer surface morphology is demonstrating via SEM observation, as well as how much the polymer surface roughness is through the AFM calculations. This paper also gives a reasonable explanation for the formation of the micro-rough surface of acrylic polymer resin with the aid of the TEM observation and DSC analysis. Finally, the effects of the crosslinker AMA content on the glass transition temperature, film transmittance, and thermal degradation stability are discussed in detail.

## 2. Experimental

### 2.1. Materials

Butyl acrylate (BA), allyl methacrylate (AMA), and tert-butyl methacrylate (t-BMA) were purchased from Shanghai Aladdin Reagent (Shanghai, China). Potassium persulfate (KPS) and sodium lauryl sulfonate (SLS) were purchased from Sinopharm Chemical Reagent (Shanghai, China). All of the reagents were of analytical grades and used as received. Mesh screen (stainless steel, 200 screening size) was purchased by AS-ONE Corporation (Tokyo, Japan). Deionized water (DI) was used for the polymerization and treatment process.

### 2.2. Preparation of Low Gloss Aqueous Acrylic Resins

**Scheme 1:**
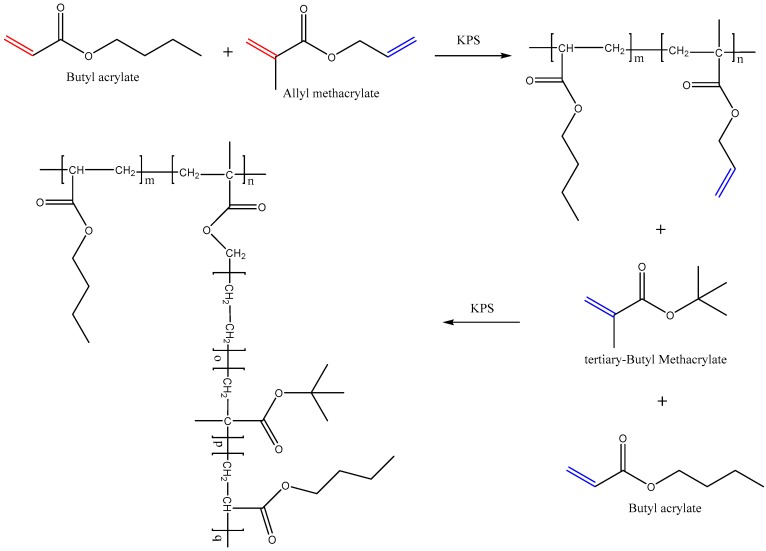
Pathway for the preparation of the acrylic polymer resins.

The composition and amount of raw materials for the preparation of aqueous acrylic resin emulsions are summarized in Table 1. First of all, deionized water and a certain amount of emulsifier SLS were charged into a 500-mL round-bottom flask under nitrogen atmosphere. It was stirred at 250 r min^−1^ with a mechanical stirrer for dissolving and dispersing. When the system temperature was heated to 70 °C, the first group of 20% BA/AMA mixed monomers (total weight percentage is fixed at 20 wt %) were dropwise added to the system for emulsifying about 40 min. Subsequently, a certain amount of initiator KPS was pumped into the system to prepare the seed emulsion within 30 min. The remaining 80% of BA/AMA mixed monomers of the first group were dropwise added to the system, followed by reacting at 70 °C for 1 h. Then, the second group of t-BMA/BA mixed monomers and the remaining KPS solution were pumped into the flask through two separate tubes at a constant rate within 30 min. Finally, the polymerization reaction continued at 70 °C for 2 h. The aqueous resins of acrylic polymer were obtained after being filtered through a 200-mesh screen. The pathway for the preparation of the acrylic polymer resins could be seen in Scheme 1.

### 2.3. Preparation of Low Gloss Aqueous Acrylic Films

The low gloss polymer films were prepared by coating the aqueous acrylic resins onto a glass sheet with a bar coater. The thickness of the wetting films was fixed at 100 μm. The coated wetting films were dried at ambient temperature for 24 h and then further dried in a convection oven at 90 °C for 1 h to remove the residual water. Finally, the polymer films were peeled off from the glass sheet and kept in a vacuum desiccator prior to testing.

### 2.4. Characterization

#### 2.4.1. Attenuated Total Reflection-Fourier Transform Infrared Spectroscopy (ATR-FTIR)

The infrared spectrum of the acrylic polymer resin was obtained on a Bruker VERTEX70 FTIR spectrometer (Bremen, Germany) under N_2_ protection. The sample was scanned 16 times at a resolution of 1 cm^−1^ over the frequency range of 4000–600 cm^−1^.

#### 2.4.2. Nuclear Magnetic Resonance (NMR)

^1^HNMR and ^13^CNMR spectra of the low gloss aqueous acrylic resin film were recorded on a Bruker DRX-400 spectrometer (Bremen, Germany). The solvent was CDCl_3_. Tetramethylsilane was used as the internal reference.

#### 2.4.3. Scanning Electron Microscopy (SEM)

The surface morphology of the tilted acrylic resin films was observed using a Zeiss-Merlin SEM (Jena, Germany) at 60° incident angle. The dried films were stained on a copper grid. Subsequently, gold powder was sprayed onto the films so as to enhance the film conductivity.

#### 2.4.4. Atomic Force Microscopy (AFM)

The AFM measurements were carried out at room temperature using a Bruker Multimode 8-HR atomic force microscope (Bremen, Germany) with a 50 μm × 50 μm scanning area and a 10 μm vertical range. All images were obtained under the ScanAsyst^®^ mode with a pixel resolution of 512 × 512. The resulting images were further processed and analyzed with NanoScope Analysis 1.8 software.

*S_a_* is the arithmetic average of the absolute values of the surface height deviations measured from the mean plane and is expressed as [22]:

(1) $$S_{a}=\underset{a}{\iint }\left|Z\left(x,y\right)\right|d\left(x\right)dy$$

*S_q_* is the root mean square average of height deviations taken from the mean image data plane and is expressed as:

(2) $$S_{q}=\underset{a}{\iint }{\left(Z\left(x,y\right)\right)}^{2}d\left(x\right)dy$$

Meanwhile, *S_z_* denotes the maximum vertical distance between the highest and lowest data points in the image.

#### 2.4.5. Differential Scanning Calorimetry (DSC)

DSC was performed using a Mettler Toledo DSC821 instrument (Küsnacht, Switzerland) to characterize the thermal response property of the low gloss acrylic films. DSC traces were recorded between −70 and 120 °C in a purified nitrogen cell and at a heating rate of 20 °C min^−1^. The values of *T*_g_ were taken as midpoint temperatures.

#### 2.4.6. Transmission Electron Microscopy (TEM)

The particle size and particle morphology of the aqueous acrylic resin were observed by TEM (JEM-2100F, Tokyo, Japan) with a 100 kV electron beam. The acrylic resin was diluted to 500 ppm with deionized water containing 2% phosphotungstic acid. Micro-copper grid was dipped into the diluted resin for a while and then taken out for drying at ambient temperature overnight before testing.

#### 2.4.7. Ultraviolet-Visible Spectroscopy (UV-Vis)

The transmittance of the low gloss acrylic films was measured via a UV-Vis (UV-2500, Shimadzu Corp., Kyoto, Japan) spectrophotometry. Tape was used to stick the films on the sample pool. The scanning wavelength ranged from 300 to 800 nm with a medium speed of scanning.

#### 2.4.8. Thermal Gravimetric Analysis (TGA)

Thermal stability of the low gloss acrylic films was evaluated with a PerkinElmer analyzer (Waltham, MA, USA) under a nitrogen flow (10 cm^3^·min^−1^). The samples were heated from 35 to 600 °C at a heating rate of 10 °C·min^−1^. The weight losses and the first derivative weights were recorded as a function of the temperature.

#### 2.4.9. Gloss Performance

The specular gloss of the acrylic films was determined at 60° incident angle using a KGZ-60 gloss-meter (Tianjin, China) according to ASTM D2457. The gloss level was tested five times and the average gloss value was reported in this paper.

#### 2.4.10. Physical and Mechanical Properties

The flexibility of the acrylic polymer films was determined with a conical mandrel apparatus (QTY-32 bending tester, Dongguan Maike Co., Ltd., Dongguan, China) according to ASTM D522. The abrasion resistance was performed on a Taber Abraser (Taber Industries, CS-17 wheels, 1000 g) according to ISO 7784.2-97 and their hardness with the pencil test was determined according to ASTM D3363. The thickness was determined using a Vernier Caliper in accordance with ISO 2808-2007. A crosscut adhesion was measured according to ASTM D3359-87. The water resistance of the acrylic films was assessed and a water absorption rate of 5% was used as a measure of the difference between good and poor water resistance. The impact resistance in accordance with GB/T 1732-93, as well as the touch feeling of the acrylic films were tested. Finally, the solid content (according to GB 1725-79) and stored stability (according to GB 6753.3-86) of the acrylic polymer resins were also evaluated.

## 3. Results and Discussion

### 3.1. Structural Characterization

**Figure 1:**
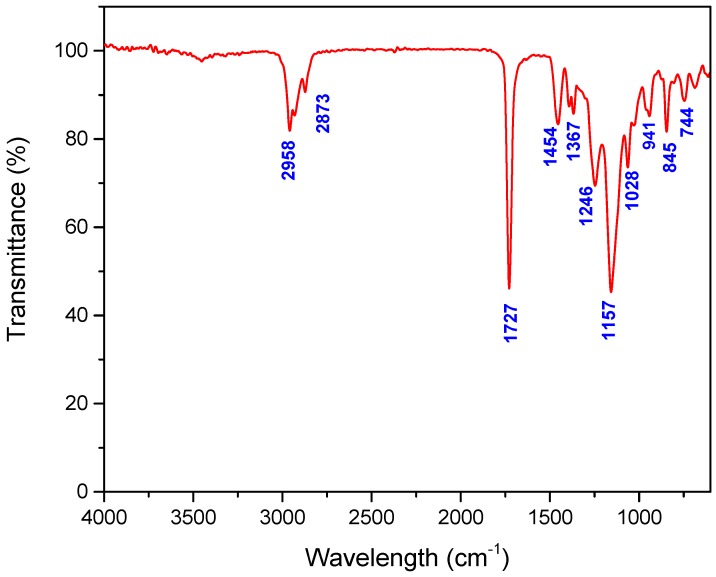
ATR-FTIR spectrum of the low gloss acrylic film with crosslinker AMA content 0.2 wt %.

Figure 1 shows the ATR-FTIR spectrum of the low gloss acrylic film with crosslinker AMA content 0.2 wt %. The band at 2800–3000 cm^−1^ was attributed to the –CH_2_– and –CH_3_ stretching vibrations of the acrylate polymer. Meanwhile, the sharp and strong peak at 1727 cm^−1^ was assigned to the –C=O stretching vibrations of the acrylate polymer. It is notable that vinyl-monomers, including the AMA crosslinker, have participated in the chemical reaction completely during the process of the emulsion polymerization. This was demonstrated by an absence of the band at 1630 cm^−1^ which was normally assigned to the –C=C– stretching vibrations [23]. The sharp absorption at 1157 cm^−1^ was ascribed to the O=C–O–C– stretching vibrations of the acrylate polymer formed. The peaks at 1454 cm^−1^ and 1367 cm^−1^ were corresponding to the –CH_2_– and –CH_3_ deformation vibrations, respectively. In general, the low gloss aqueous acrylic polymer resin was prepared successfully.

**Figure 2:**
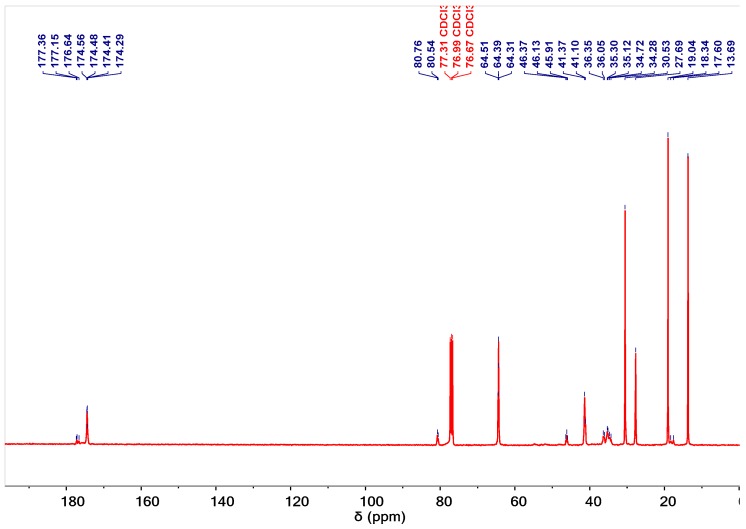

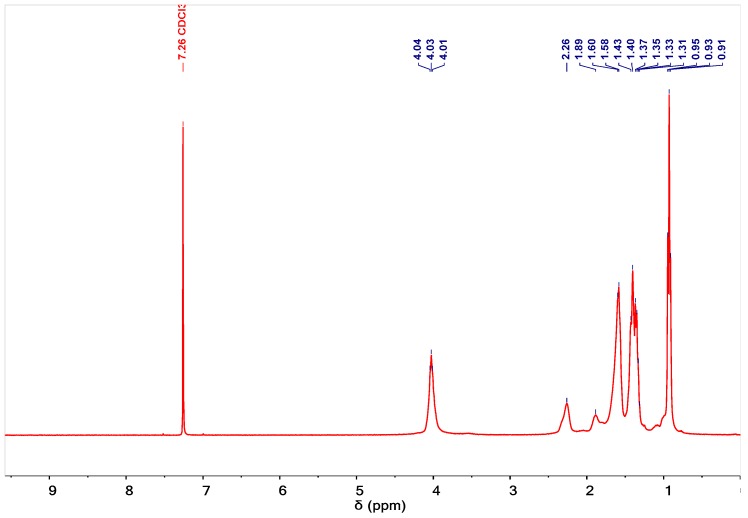
^1^HNMR and ^13^CNMR spectra of the low gloss acrylic resin with crosslinker AMA content 0.2 wt % in CDCl_3_.

Figure 2 shows the ^1^HNMR and ^13^CNMR spectra of the low gloss acrylic resin with crosslinker AMA content 0.2 wt %. In the ^13^CNMR spectrum, there were no peaks at about 120–130 and 160 ppm, which were respectively attributed to –C=C– vinyl and O=C–O–R carbonyl carbons of the acrylic monomers. The newly emerged peaks at 177.35 ppm and the peaks at 80.76 and 27.69 ppm were corresponding to the t-BMA groups after polymerization. Similarly, the newly emerged peaks at 174.41 ppm and 64.51 ppm were ascribed to the O=C–O–C–R resonance of the BA and AMA after being polymerized. With regard to the ^1^HNMR spectrum, there were no signals in the range of 5–7 ppm, suggesting that all of the –CH=CH– vinyl groups of the acrylic monomers had participated in the polymer process. The signal observed at 4.03 ppm was assigned to the proton of O=C–O–CH_2_–R groups. All in all, these results demonstrate that all of the acrylic monomers have copolymerized to form the acrylate co-polymer.

### 3.2. Particle Morphology Observation

**Figure 3:**
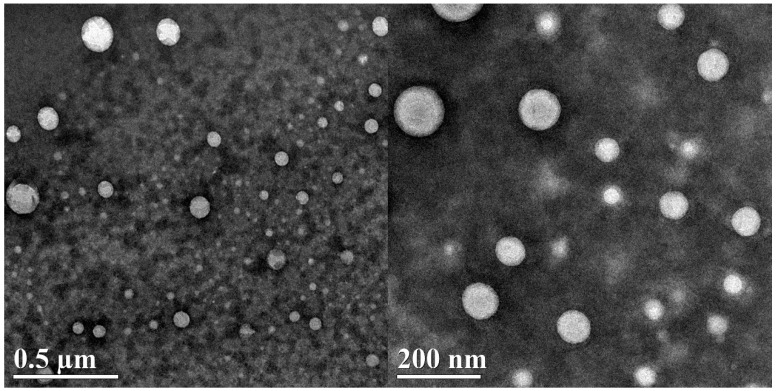
TEM images of the low gloss aqueous acrylic resin with crosslinker AMA content 0.2 wt % at different magnifications.

Figure 3 shows the TEM images of the low gloss acrylic resin with crosslinker AMA content 0.2 wt % at two different magnifications. It can be seen that all of the aqueous acrylate polymer emulsion particles were spherical in shape. The particle diameter of the emulsion was varied, ranging from tens of nanometers to hundreds of nanometers with most ranging between 20 and 200 nm. This wide distribution of the emulsion particle diameter was due to the addition of the crosslinker AMA and the two-stepped emulsion polymerization. The emulsion particles with a smaller particle diameter could be attributed to the lower molecular weight acrylate polymer that did not participate in the cross-linking reaction. Likewise, the larger particles can be assigned to the higher molecular weight acrylate polymer particles that have finished the cross-linking reaction under the involvement of the crosslinker AMA. This can be verified from the observation of two glass transition temperatures in the DSC curve. In general, these emulsion particles with a greatly wider particle diameter distribution developed mechanical instability during the film formation process, thus yielding a micro-rough film surface. When the incident light was cast on this rough polymer film, most incident light experienced absorption, refraction, and multiple reflections and little reflected light was received, leading to a low gloss effect of the acrylic film [24,25].

### 3.3. Film Surface Analysis

**Figure 4:**
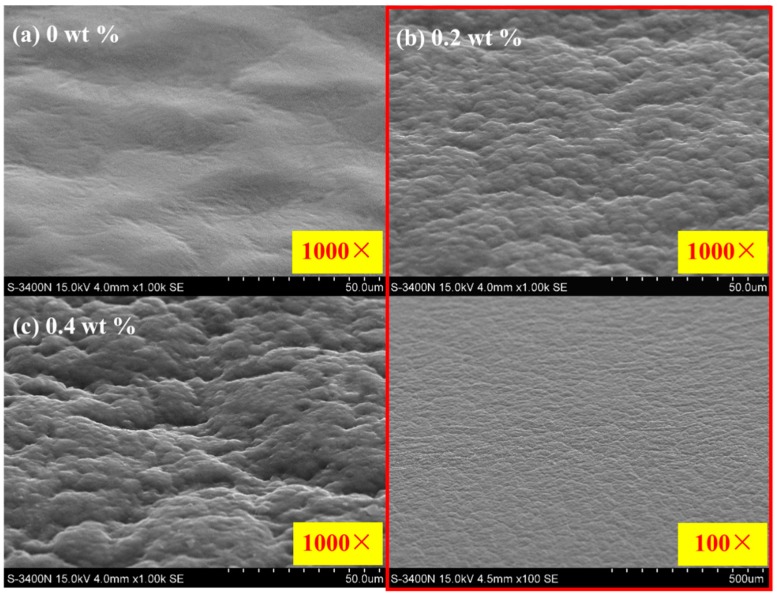
The images of the tilted low gloss acrylic polymer films at 60° angle with crosslinker AMA contents ranging from (**a**) 0 wt %, 1000× to (**b**) 0.2 wt %, 1000× or 100× and to (**c**) 0.4 wt %, 1000×, respectively.

It is acknowledged that the tilted samples are much easier to observe the rough surface morphology. For this reason, SEM was used to observe the tilted samples at 60° incident angle and the resulted images could be seen from Figure 4. In Figure 4a, when the crosslinker AMA content was 0 wt %, the film surface was prone to be flat and smooth. With the increase of the crosslinker AMA content from 0 to 0.2 wt %, and further up to 0.4 wt %, the film surfaces became to be much rougher. Especially in Figure 4c, when the crosslinker AMA content was 0.4 wt %, some small voids and rugged areas were visible. In general, the low gloss aqueous acrylic resin with crosslinker AMA content 0.2 wt % demonstrated better film formation property. When the sample with crosslinker AMA content 0.2 wt % was observed at a relatively low magnification (100×), the film surface was very exquisite. This illustrates the reason why this low gloss acrylic film with crosslinker AMA content 0.2 wt % was smooth when it was seen with the naked eyes. Unlike the conventionally dulled coatings (using silica or organic matting agents) having a rather discontinuous and multi-seeded appearance, the morphology associated with this low gloss acrylic film with crosslinker AMA content 0.2 wt % was continuous and flexible, which was capable of providing delicate touch feeling. Thus, this acrylic polymer resin through controlling the film morphology to achieve the self-matting effect showed greater advantages than the traditional low gloss resins by adding the matting agents.

### 3.4. Surface Roughness

**Figure 5:**
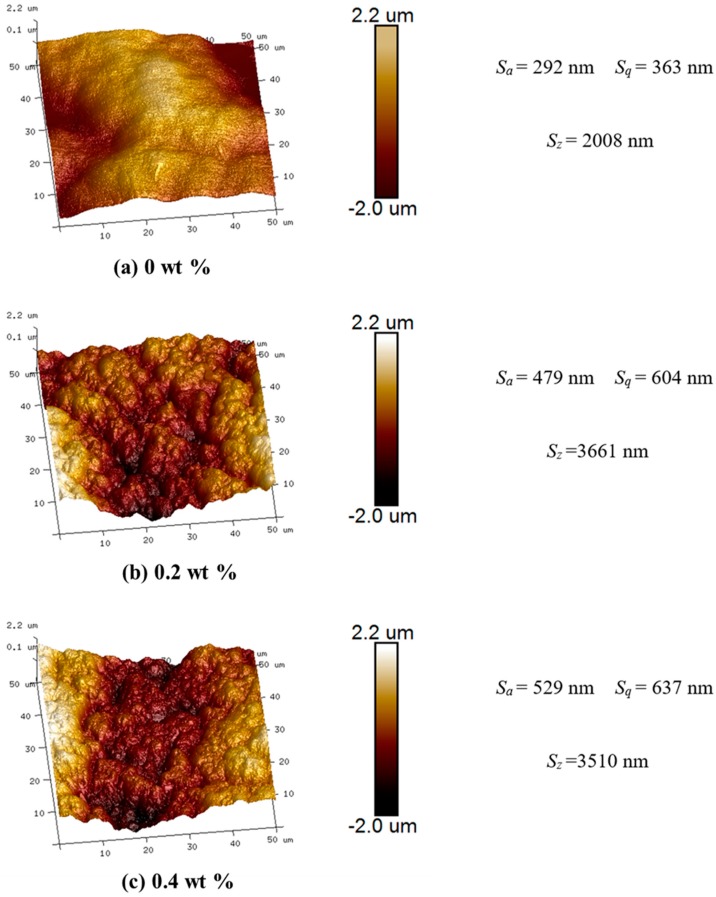
Three-dimensional topographies and surface roughness of the low gloss acrylic polymer films with crosslinker AMA contents ranging from (**a**) 0 wt % to (**b**) 0.2 wt % and to (**c**) 0.4 wt %, respectively.

It is necessary for matting coatings to measure the surface roughness of films as it affects the visual impressions of the coated products and people’s touch feelings [26]. The visual impression of a surface is recognized as various types of glossiness that are generally classified as different degrees of gloss. Among these, specular gloss is the most frequently used parameter for evaluating coatings [27,28,29]. The specular gloss measures the intensity of mirror-like reflection, i.e., when the angle of incidence is equal to the angle of reflection [30]. The more light that is reflected in this way, the greater the impression of gloss is [31,32]. Smooth surfaces reflect a major part of the light in this way. On rough surfaces, the light is scattered in several directions [33]. Therefore, the image forming quality is diminished and the reflected image is blurred. In Figure 5, the acrylic film with no crosslinker AMA showed a relatively smooth surface. The arithmetical average roughness of *S_a_* of this film was 292 nm and the root mean square roughness of *S_q_* was 363 nm. The maximum vertical distance between the highest data point and the lowest data point was only 2008 nm. These roughness parameters of *S_a_* and *S_q_* were smaller than the wavelength of visible light 400–800 nm, which made it hard to scatter the visible light, leading to a glossy surface. The latter two aqueous acrylic films showed higher surface roughness levels, and the difference in rough levels was slight. Both *S_a_* values were around 500 nm. This roughness fell just within the wavelength range of visible light, and thus they were capable of scattering the light, leading to a matt film [34,35].

### 3.5. DSC Analysis

**Figure 6:**
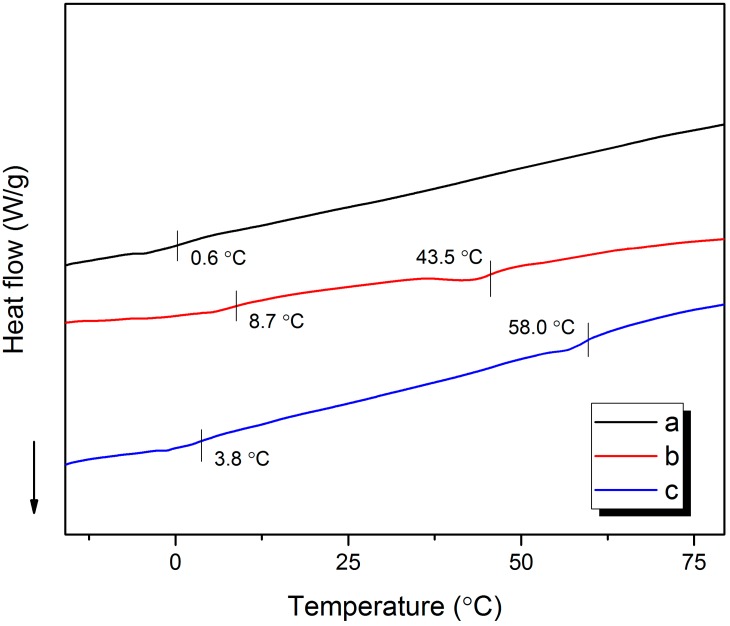
DSC curves for the low gloss acrylic polymer films with crosslinker AMA contents ranging from (**a**) 0 wt % to (**b**) 0.2 wt % and to (**c**) 0.4 wt %, respectively.

Figure 6 shows the effect of AMA crosslinker content on the glass transition temperature (*T*_g_) of the acrylic films. The acrylic film with zero addition of AMA crosslinker, that is curve (a), exhibited only one glass transition temperature (*T*_g_) of 0.6 °C, which could be the *T*_g_ of the copolymer of BA and t-BMA. In contrast, both curve (b) and curve (c) exhibited two *T*_g_ values_,_ which were ascribed to the addition of the AMA crosslinker. The lower *T*_g_ value corresponded to the copolymer of BA and t-BMA, while the higher *T*_g_ value was ascribed to the copolymer of BA, AMA, and t-BMA. It should also be observed that the lower *T*_g_ value of 3.8 °C in curve (c) was smaller than the lower *T*_g_ value of 8.7 °C in curve (b), and, on the contrary, the higher *T*_g_ value of 58.0 °C in curve (c) was greater than the higher *T*_g_ value of 43.5 °C in curve (b). This result could be explained by the fact that an increase in AMA crosslinker content led to an increase in the product of primary polymerization containing AMA crosslinker and, therefore, the second polymerization naturally needed more t-BMA to participate in the reaction. Therefore, the higher *T*_g_ value increased with increasing the AMA crosslinker content. Due to the decrease of remaining AMA crosslinker monomer, the *T*_g_ of copolymer between the remaining BA and t-BMA also decreased. In conclusion, the significant difference between the two *T*_g_ values of the acrylic films might lead to the difference in curing order during the drying process. The curing order difference probably caused surface instability of films, leading to the formation of self-rough surfaces [36,37,38].

### 3.6. Film Transparency

**Figure 7:**
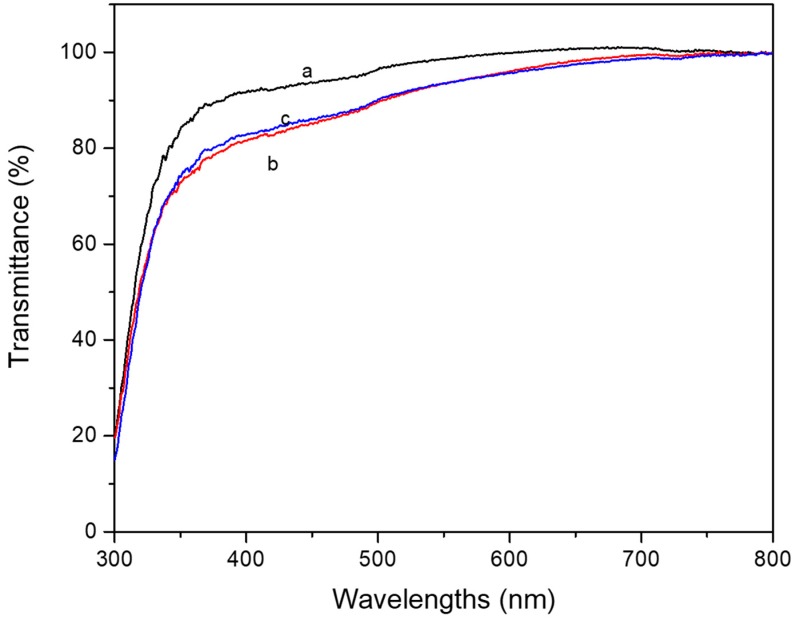
UV-Vis spectra of low gloss acrylic polymer films with crosslinker AMA contents ranging from (**a**) 0 wt % to (**b**) 0.2 wt % and to (**c**) 0.4 wt %, respectively.

As shown in Figure 7, the film transparency of the low gloss acrylic films was measured via UV-Vis spectra in the transmittance mode. In general, all of the samples were hardly capable of transmitting the light below the wavelength of 400 nm. In the wavelength range of visible light of 400–800 nm, they seemed to have good transmittance abilities. It is clearly evident in Figure 7 that the low gloss acrylic film without addition of the AMA crosslinker, labeled as curve (a), possessed the highest transmittance (approximately 95%), while the transmittances of curves (b) and (c) were very close to one another (approximately 85%). This result indicates that the addition of the AMA crosslinker led to the decrease of the film transparency to some extent. However, the further increment of the AMA crosslinker content did not significantly change the film transparency. Even though the acrylic polymer film without crosslinker (curve (a)) embraced up to 95% transmittance, the film surface was flat and smooth and it had a poor matting effect. In contrast, the acrylic films with the addition of the AMA crosslinker (see curves (b) and (c)) were able to provide both superior film transparency and low gloss effect, which made them suitable for extinction in leather applications.

### 3.7. Thermal Stability Analysis

**Figure 8:**
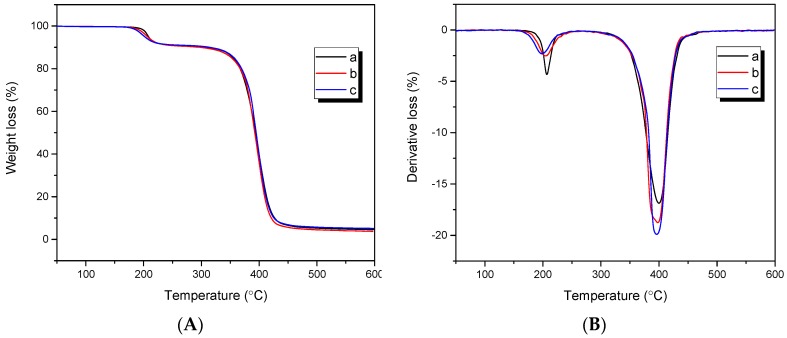
TGA (**A**) and DTG (**B**) curves of the low gloss acrylic polymer films with crosslinker AMA contents ranging from (a) 0 wt % to (b) 0.2 wt % and to (c) 0.4 wt %, respectively.

Based on the TGA and DTG curves shown in Figure 8, all of the low gloss aqueous acrylic films with the crosslinker AMA contents ranging from 0 wt % to 0.2 wt % and to 0.4 wt % underwent two prominent degradation stages. The first degradation stage began at a temperature of 170 °C and ended at nearly 230 °C. This stage primarily corresponded to the release of internal water, residual acrylic monomers, and some oligomers. The second decomposition stage began at around 320 °C and decomposed at approximately 450 °C. This stage corresponded to the decomposition of the backbone chains of the acrylic polymers. In general, the effect of the AMA crosslinker content did not exhibit a significant influence on thermal stability. It should also be observed that during the first degradation stage with an increase of the AMA crosslinker content, the temperature of weight loss of acrylic films started earlier but the rate of weight loss was much slower, and, finally, the remaining weight of acrylic films reached the same level (about 90%). In the second decomposition stage, when the degradation temperature was below 400 °C, the rate of weight loss decreased with an increase of the AMA crosslinker content. This indicates that due to the increase of AMA crosslinker content, the thermal stability of acrylic films increased slightly. Further, the maximum degradation rate of acrylic films occurred at 400 °C, showing excellent thermal degradation stability.

### 3.8. Comprehensive Properties

Table 2 shows the physical and mechanical properties of the aqueous acrylic resins with crosslinker AMA contents ranging from 0 wt % (a) to 0.2 wt % (b) and to 0.4 wt % (c), respectively. The solid content of all the acrylic resins was 25%. It should be noted that the 60° specular gloss of the acrylic film with no addition of AMA crosslinker was up to 78.0 gloss units, whereas in contrast, the gloss of the acrylic polymer film with the addition of AMA crosslinker 0.2 wt % decreased sharply to 4 gloss units, providing an excellent matting effect. All three samples had the best grade flexibility of 2 mm. The abrasion resistance and pencil hardness of the acrylic films increased with increasing the AMA crosslinker content, but the adhesion decreased slightly. These results attributed to the increase of the cross-linked network structure in the polymer molecules. However, the acrylic film with 0 and 0.2 wt % crosslinker AMA provided soft-touch feelings, whereas the film with 0.4 wt % crosslinker AMA showed hard-touch feeling. In addition, the water resistance of the acrylic film with zero addition of crosslinker AMA was poor while the low gloss acrylic films with an addition of 0.2 and 0.4 wt % were good. As a result, the low gloss acrylic film with 0.2 wt % crosslinker AMA demonstrated the best comprehensive physical and mechanical properties when considering all the aspects for practical usage.

## 4. Conclusions

In this study, we described a novel approach to fabricate an aqueous acrylic resin with low gloss and high transparency via controlling the film morphology. The strategy was based on the synthesis of a novel film-forming acrylic polymer resin that gave rise to a self-rough surface morphology at sub-micron roughness levels. The formation of the micro-roughed surface of the aqueous acrylic resin was achieved by controlling the amount and addition order of each monomer, as well as the introduction of the cross-linking monomer AMA, through which incompatibility behavior within the resultant resin could be caused. The results show that this aqueous acrylic resin without the addition of extraneous matting agent could arrive at less than 4 gloss units at 60° incident angle and up to 85% light transmittance after being dried upon air or oven. In general, this low gloss aqueous acrylic polymer film showed good abrasion resistance and soft-touch feeling, as well as excellent thermal stability.

## Figures and Tables

**Table 1:** The composition and amount of raw materials for preparing the low gloss aqueous acrylic resins.

Component	Abbr.	Composition (wt %)
Monomer	BA	19.6–20
	AMA	0–0.4
	t-BMA	4.8
	BA	0.2
Emulsifier	SD	0.075
Initiator	KPS	0.15
Medium	DI	75

**Table 2:** Comprehensive properties of the acrylic resin with crosslinker AMA contents ranging from 0 wt % (a) to 0.2 wt % (b) and to 0.4 wt % (c), respectively.

Type of Test	0 wt %	0.2 wt %	0.4 wt %
Solid content (%)	25	25	25
60° specular gloss (°)	78.0	4.0	3.2
Flexibility (mm)	2	2	2
Abrasion resistance (/circle)	200	250	280
Adhesion	5B	5B	4B
Pencil hardness	H	2H	4H
Touch feeling	Soft-touch	Soft-touch	Hard-touch
Water resistance	Poor	Good	Good
Impact resistance (500 g)	Good	Good	Good
Storage stability (180 days)	Good	Good	Good
